# Characterization and Propagation of Tumor Initiating Cells Derived from Colorectal Liver Metastases: Trials, Tribulations and a Cautionary Note

**DOI:** 10.1371/journal.pone.0117776

**Published:** 2015-02-06

**Authors:** Mark I. James, Lynne M. Howells, Ankur Karmokar, Jennifer A. Higgins, Peter Greaves, Hong Cai, Ashley Dennison, Matthew Metcalfe, Giuseppe Garcea, David M. Lloyd, David P. Berry, William P. Steward, Karen Brown

**Affiliations:** 1 University of Leicester, Leicester, United Kingdom; 2 University Hospitals of Leicester, Leicester, United Kingdom; 3 University Hospitals of Wales, Cardiff, United Kingdom; University of Pittsburgh School of Medicine, UNITED STATES

## Abstract

Tumor initiating cells (TIC) are increasingly being put forward as a potential target for intervention within colorectal cancer. Whilst characterisation and outgrowth of these cells has been extensively undertaken in primary colorectal cancers, few data are available describing characteristics within the metastatic setting. Tissue was obtained from patients undergoing surgical resection for colorectal liver metastases, and processed into single cell suspension for assessment. Tumor initiating cells from liver metastases were characterised using combinations of EPCAM, Aldehyde dehydrogenase activity, CD133 and CD26. CD133 expression was significantly lower in patients who had received chemotherapy, but this was accounted for by a decrease observed in the male patient cohort only. ALDH^high^ populations were rare (0.4 and 0.3% for EPCAM^+^/ALDH^high^/CD133^-^ and EPCAM^+^/ALDH^high^/CD133^+^ populations respectively) and below the limits of detection in 28% of samples. Spheroid outgrowth of metastatic tumor cells across all samples could not be readily achieved using standard spheroid-formation techniques, thus requiring further method validation to reliably propagate cells from the majority of tissues. Spheroid formation was not enhanced using additional growth factors or fibroblast co-culture, but once cells were passaged through NOD-SCID mice, spheroid formation was observed in 82% samples, accompanied by a significant increase in CD26. Order of spheroid forming ability was ALDH^high^>CD133>CD26. Samples sorted by these markers each had the ability to reform ALDH^high^, CD133 and CD26 positive populations to a similar extent, suggestive of a high degree of plasticity for each population. Ex vivo TIC models are increasingly being utilised to assess efficacy of therapeutic interventions. It is therefore essential that such investigations use well-characterised models that are able to sustain TIC populations across a large patient cohort in order that the inherent heterogeneity observed in cancer populations is maintained.

## Introduction

In the UK, colorectal cancer (CRC) has an approximate 5-year survival rate of 55%, and a 10-year survival of 51%. These statistics are dramatically altered depending on whether the patient presents with localised disease (5-year survival of 90%) or metastatic disease (5 year survival of 12%) [1].

Tumor initiating cells (TICs) or cancer stem-like cells (CSCs) are increasingly being put forward as a potential target for intervention within the colorectal cancer setting. In CRC, the evidence for the existence of a TIC population is compelling, although the origin of these cells has yet to be definitively established, and consequently, they shall be referred to as TICs rather than CSCs. Notwithstanding this, a potential candidate for their origin in CRC is that of a transformed adult stem cell, with evidence to support this hypothesis in humans, arising from studies of patients with Familial Adenomatous Polyposis Coli (FAP), where tumor overpopulation has been shown to arise from mutated cells at the base of the crypts [2,3].

Within the context of CRC, no markers (either alone or in combination) have yet been found to completely and confidently identify the TIC populations within a tumor. The first TIC marker reported for CRC was CD133 [4], but further investigations have cast doubt on whether it can be utilised singularly as a definitive marker [5–7]. The ability of CD133^+^ cells to form tumors appears to be enhanced when present in combination with other TIC markers, such as activity of the enzyme Aldehyde dehydrogenase 1 (ALDH1) [8]. High ALDH1 activity has been associated with TIC populations in breast cancer, lung cancer, and acute myeloid leukaemia [9–11]. In normal colonic crypts, ALDH1A1 protein expression is very specific to a small population of cells at the base of crypts, with a sub-population of these cells also CD133^+^, and so represents a good TIC marker candidate in human CRC [8]. Initially the hierarchal model was believed to be rigid with only the TICs forming the apex from which all the other tumours cells were derived. However, this concept has recently been challenged, in that more differentiated cells may also have the capacity to form TICs under the correct setting, with the microenvironment playing an integral part in regulating stem-like characteristics [12].

The majority of investigations involving TICs have focused on primary cancers, rather than on TICs derived from the metastasic site. There is still much ambiguity as to whether metastatic TICs can be defined in a similar manner to those cells derived from the primary tumor site, or whether addition of further markers may help to better define this population. Increasingly, it has been hypothesised that there may be a metastatic TIC, which maintains a phenotype distinct from that of the primary tumor TIC [13,14]. This metastatic TIC is proposed to derive from a migratory TIC [15]. Alternatively, the metastatic niche itself may dictate and be responsible for, maintenance of differential marker expression in resident TICs [16].

There is much value in the incorporation of TIC models in to *in vitro* and *ex vivo* investigations for colorectal cancer. It is generally accepted that tumor cells in monolayer culture are of rather limited use in the preclinical testing of novel anticancer interventions. Increasingly, 3D tissue culture models are being employed for the selection of potential anti-tumour agents, as they can mimic aspects of *in vivo* tumor hierarchy with greater fidelity than cells in monolayer [17–19]. Recently, spheroid culture systems have been shown to allow maintenance of a cancer cell population with TIC properties [20]. These systems are of particular interest in preclinical testing as there is increasing evidence to support the notion that the effects of novel, potentially useful interventions on TICs could be more predictive of anti-tumor efficacy *in vivo* than their effects on differentiated cells [13,21,22]. Numerous 3D culture techniques have been described for the propagation of TICs across a wide variety of human tumors, and have been validated with appropriate dose-limiting dilutions using spheroid-forming cells in NOD-SCID mouse models.

Spheroid models of human primary colorectal cancers for assessment of TICs have been well established [4]. These consist most often of a simple liquid overlay/low adherence system, which typically yield spheroids for 40–50% of samples (8,9). However, the role of TICs in metastasis remains less well understood, coincident with the fact that very few models of colorectal metastasis exist. This is likely due to the technical challenges associated with maintenance of primary colorectal liver metastasis (CRLM) cultures.

Primary aims of this study were thus: to develop a CRLM model that could be readily propagated for use in drug intervention studies; to assess effects of external influences on TIC-like properties of CRLM-derived cells; to assess plasticity under adherent culture conditions. Overall, this study contributes to the body of knowledge which may ultimately allow *ex vivo* assessment of TIC-targeting treatment options in late stage CRC.

## Materials and Methods

Aldefluor assay kit was obtained from Stemcell Technologies (Grenoble, France). PE-conjugated Epithelial Cellular Adhesion Molecule (EpCAM) and APC-conjugated CD133-AC133 were from Miltentyi Biotech (Surrey, UK). FITC-conjugated CD26 was from BD Biosciences (Oxford, UK). 18Co cells were from ATCC (Middlesex, UK). Antimycotic/antibiotic cocktail (containing penicillin, streptomycin, fungizone) B-27 supplement, DMEM/F12 medium (1:1) Hyclone, Epithelial Growth Factor (EGF), Fibroblast Growth Factor (FGF), N-2 supplement and Neurobasal medium were all obtained from Fisher Scientific (Leicestershire, UK) and Collagenase Type 4 from Worthington Biochemical Corp (New Jersey, USA). Media 199, Hanks Balanced Salt Solution (HBSS), Foetal Calf Serum (FCS) and Trypsin/EDTA (T/E) were from Invitrogen Ltd, (Paisley, UK) and Matrigel from BD Biosciences (Oxford, UK). The pluripotent Stem Cell Proteome Profiler kit, R-spondin and Wnt-3a were supplied by R&D Systems (Oxford, UK), and all other reagents were from Sigma (Dorset, UK).

Tumor samples were obtained from 65 patients undergoing surgical resection for colorectal liver metastases at University Hospitals of Leicester NHS Trust, with 50 of these suitable for inclusion for flow cytometric analysis. Samples were collected immediately following surgery and placed in ice-cold media 199 prior to processing within one hour. Ethical approval for this fully anonymised, excess tissue study was granted by Leicestershire, Northamptonshire and Rutland ethics committee (REC reference 09/H0402/45).

Tumor tissues were received into the laboratory and processed immediately. In brief, tumor tissue was finely minced on ice in media 199, collagenase added (2000 units/mL) and the sample incubated using a MACSmix tube rotator (Miltenyi Biotech) at 37°C for 30–40 minutes. Cells were pelleted at 350 x g, re-suspended in fresh media 199 and passed through a 100 μm followed by a 40 μm filter, prior to being washed twice in HBSS. Cells were counted and viability assessed using trypan blue before cells were appropriately aliquoted for experimental procedures. One aliquot of cells from each sample was always reserved for flow cytometric assessment of stem-like components, and a variety of culture methods assessed.

Flow cytometry was performed on single cell suspensions derived from patient tissue or disaggregated spheres using a FACS Aria II (Becton Dickinson, Oxford, UK). Stem-like and epithelial components were characterised using EPCAM, CD133, CD26 and aldehyde dehydrogenase activity.

### Pluripotent stem cell protein profiler array

Samples were sorted (purity) into EpCAM positive populations using the BD FACS Aria II, the cells lysed, and the proteome profile determined using the Proteome Profiler Array Kit (R&D Systems) as per the manufacturer’s instructions. Array membranes were analysed and quantified using the Syngene Chemigenius II system (Cambridge, UK)

### Low adherence culture

Cells (30,000) were seeded on to low attachment plates (Appleton Woods, Birmingham, UK) at 37°C, 10% CO_2,_ in stem cell media containing 50% neurobasal medium, DMEM/F12 medium 1:1 Hyclone, 1% N-2 Supplement, 2% B-27 Supplement, 2% Antibiotic-antimycotic, 2 ng/mL Heparin, 20 ng/mL EGF and 20 ng/mL FGF-2. Media was supplemented with 500 μL of fresh stem cell media once per week.

### Additional growth factors

In order to facilitate sphere formation, further growth factors were incorporated in to standard stem cell medium. These included R-spondin (1 μg/mL), Wnt-3a (100 ng/mL) and Hepatocyte Growth Factor (20 ng/mL).

### Matrix embedding

Matrigel was allowed to thaw overnight at 4°C, and kept on ice during use. Matrigel was mixed 1:1 with 30,000 tumour-derived cells in stem cell media and aliquoted in to 6-well plates to solidify. Standard stem cell media was used for subsequent culture of matrigel-embedded cells.

### Fibroblast co-culture (conditioned media)

18Co cells (750,000) were seeded in a 75 cm^2^ flask for 24 hours in their standard media (low glucose DMEM with 1% glutamax and 10% FCS). Cells were then washed twice with 10 mL of PBS and incubated for 24 hours with 10 mL of stem cell media without EGF and FGF. The conditioned stem cell media was subsequently aspirated, cleared of any debris by centrifugation, and then mixed 1:1 with normal stem cell media.

### Fibroblast co-culture (non-contact method)

18Co cells were seeded at 15,000 cells/well on a 6-well plate in their standard media and left to adhere overnight prior to replacing with 3 mL stem cell media. A 1 μm polyethylene terephthalate membrane tissue culture insert (BD biosciences) was placed in to the well and a further 2 mL of stem cell media placed in to the top of the insert containing 30,000 tumour-derived cells, allowing diffusible growth factors to pass through the membrane.

### Fibroblast co-culture (direct contact method)

18Co cells were seeded on to 6-well plates for adherent growth at 20,000 cells/well in 2 mL of their standard media, and left to attach for three days (5% CO_2_, 37°C). Subsequently, cells were washed with PBS, and 2 mL of stem cell media added which contained a total of 30,000 tumour-derived cells. Cells were co-cultured in 10% CO_2_ (37°C).

For all culture methods, spheroid growth was assessed every 7 days for 28 days using a Nikon (Surrey, UK) TE 2000 U camera system with Eclipse software.

Animal studies were carried out under animal project license PPL 80/2167, granted to Leicester University by the United Kingdom Home Office. The experimental design was vetted by the Leicester University Local Ethical Committee for Animal Experimentation and met the standards required by the UKCCR for animal welfare. NOD-SCID mice (NOD.CB17/JHliHsd-Prkdcscid, Harlan, UK) were anesthetized by exposure to isofluorane and tissue samples (2 mm x 2 mm x 2mm) placed inside subcutaneous pockets made on both flanks. Tumor growth was monitored until the tumour reached an estimated weight equivalent to 5% of the animal’s body weight (~17 mm). At this point, tumors were serially passaged into further NOD-SCID mice. Part of the tumor tissue was collagenase digested for flow cytometric profiling and assessment of sphere formation under low adherence culture, and part formalin fixed, paraffin wax embedded, sectioned and stained with haematoxylin and eosin for conventional light microscopic characterisation.

## Results

### Patient demographics

Patient demographics are described in Table 1. Out of 50 patients, 32 (64%) were male (mean age 67.4, range 52–82) and 18 were female (mean age 67.1, range 50–83). Seventy two percent were known to be in receipt of chemotherapy, with 81% of this cohort receiving adjuvant therapy following resection of the primary colorectal tumor, and 28% receiving neo-adjuvant chemotherapy for downstaging of liver metastases. Twenty one percent of patients received more than one therapeutic intervention. The most common treatment modalities were Capecitabine (35%).and FOLFOX (38%) with 21% of FOLFOX patients receiving concurrent cetuximab/bevacizumab. Two patients underwent synchronous surgery for primary colorectal cancer and CRLM.

**Table 1:** Patient demographics.

Patient Demographics	Patient numbers
Male (age range)	32 (52–82)
Female (age range)	18 (50–83)
Patients in receipt of chemotherapy	36
Neo-adjuvant chemotherapy for primary tumours	4
Adjuvant chemotherapy for primary tumours	29
Neo-adjuvant chemotherapy for liver metastases	10
Treatment modality (%):	
FOLFOX	38
Capecitabine	35
FOLFOX +Cetuximab/Bevacizumab	21
FOLFIRI	3
CAPOX	3

### TIC characteristics of CRLM tissue

**Fig 1:**
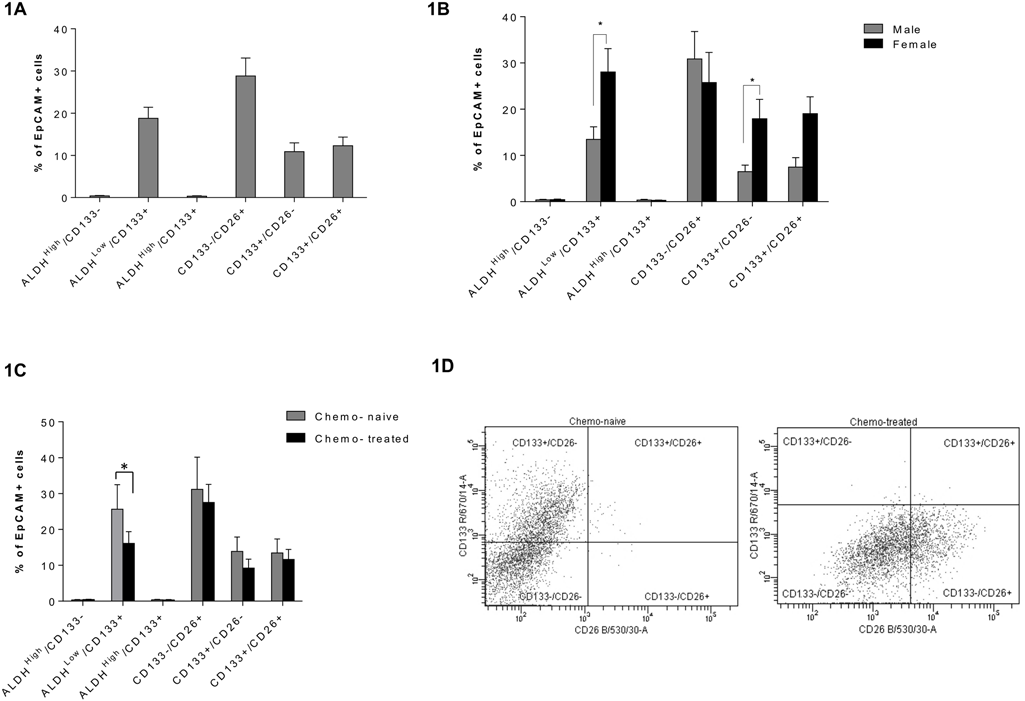
TIC marker expression in colorectal liver metastases. (A) Shows mean (±SEM) expression of TIC markers analysed by flow cytometry from patients undergoing resection for CRLM. N = 62 for ALDH combinations, N = 44 for CD26 combinations. (B) Mean (±SEM) TIC marker expression comparing males vs females. Male N = 39 and 26 for ALDH and CD26 combinations respectively, female N = 22 and 17 for ALDH and CD26 combinations respectively (independent t-tests, * = P≤0.05). (C) TIC marker expression in EpCAM^+^ cells stratified based on patient chemotherapy status. Chemo naïve N = 14, chemo-treated N = 36. (mean ± SEM, Mann Whitney-U test, * indicates P = 0.05). (D) Representative dot plot for CD26 (x-axis) and CD133 (y-axis) in a chemo-naïve (left) and chemo-treated (right) patient.

All patient samples were initially analysed by flow cytometry (Table 2) to determine the following populations as a percent of EpCAM^+^ cells (Fig. 1A): ALDH1^High^/CD133^-^ (0.4%±0.09); ALDH1^low^/CD133^+^ (18.8%±2.6); ALDH1^High^/CD133^+^ (0.35%±0.09); CD133^-^/CD26^+^ (28.8%±4.2); CD133^+^/CD26^-^ (10.9%±2.1); CD133^+^/CD26^+^ (12.28%±2.1). Significant differences were observed between males vs females for expression of CRLM TIC marker combinations incorporating CD133 (Fig. 1B), namely: ALDH^low^/CD133^+^ p = 0.004 (17.9% vs 28.1%) and CD26^-^/CD133^+^ p = 0.01 (6.5% vs 17.9%). There was no correlation between the expression of TIC markers and age, with R^2^ values being < 0.05 for ALDH, CD133, CD26 and EpCAM) (S1 Fig). When the groups were separated into those patients who were chemotherapy naïve vs those patients who had received chemotherapy (Fig. 1C/D), the ALDH^low^/CD133^+^ population exhibited a significantly lower expression in the latter cohort (25.6 vs 16.1%, p = 0.05), with this decrease accounted for almost exclusively within the male population (S2 Fig). In an attempt to further sub-categorize the TIC marker expression pattern identified by flow cytometry within this cohort, patient samples were flow cytometrically sorted using EpCAM expression (for epithelial purity), prior to being analysed for a panel of well-characterised pluripotent stem cell markers (including Oct3/4, Nanog, Sox2, AFP, GATA-4, HNF and snail) and markers of differentiation such as E-cadherin, using a proteome profiler array. There were no differences in these markers between cells containing high or low levels of EPCAM, ALDH, CD133 or CD26. Furthermore, there were no differences in proteome profile markers between chemotherapy-naïve and chemotherapy-treated patients (S3 Fig).

**Table 2:** Flow cytometry data (EpCAM^**+**^ cells only) showing TIC populations by gender and chemotherapy status.

	Male + chemo	Male chemo naive	Female + chemo	Female chemo naive
ALDH1^High^/CD133 (±SEM)	0.41(±0.15)	0.34(±0.20)	0.49(±0.19)	0.35(±0.17)
ALDH1^Low^/CD133^+^ (±SEM)	10.83(±3.16)	21.11(±4.61)	26.30(±6.93)	31.62(±6.58)
ALDH1^High^/CD133^+^ (±SEM)	0.34(±0.16)	0.40(±0.31)	0.26(±0.07)	0.38(±0.11)
CD133^-^/CD26^+^ (±SEM)	27.87(±7.06)	36.03(±11.08)	26.63(±8.63)	24.36(±11.14)
CD133^+^/CD26^-^ (±SEM)	4.83(±1.52)	9.34(±2.62)	16.50(±5.42)	20.20(±7.30)
CD133^+^/CD26^+^ (±SEM)	5.70(±2.72)	10.53(±2.82)	19.98(±4.92)	17.52(±5.87)

### Spheroid—forming ability of metastatic tissue

Standard low adherence culture proved to be poor conditions for generating spheroids from CRLM. Only four of 50 CRLM samples (8%) were able to form spheroids, defined as structures >2000B0035m diameter. Of these, not more than one sample (2%) produced spheroids that could be serially passaged. Next, several experimental procedures which have been reported to aid formation of spheroids derived from primary CRC tumors, were applied to five different patient-derived CRLM samples, in an attempt to improve spheroid formation from metastatic tissues. Incubation of cells with growth factor supplements or placement of cells within a supportive matrigel matrix to mimic microenvironmental and stromal interactions [23] did not enhance spheroid forming capacity (S4 Fig). Similarly, use of conditioned media from 18Co fibroblast cultures, or co-culture with fibroblasts using the insert system [24,25] failed to improve metastasis-derived spheroid formation rate in 5/5 CRLM samples.

**Fig 2:**
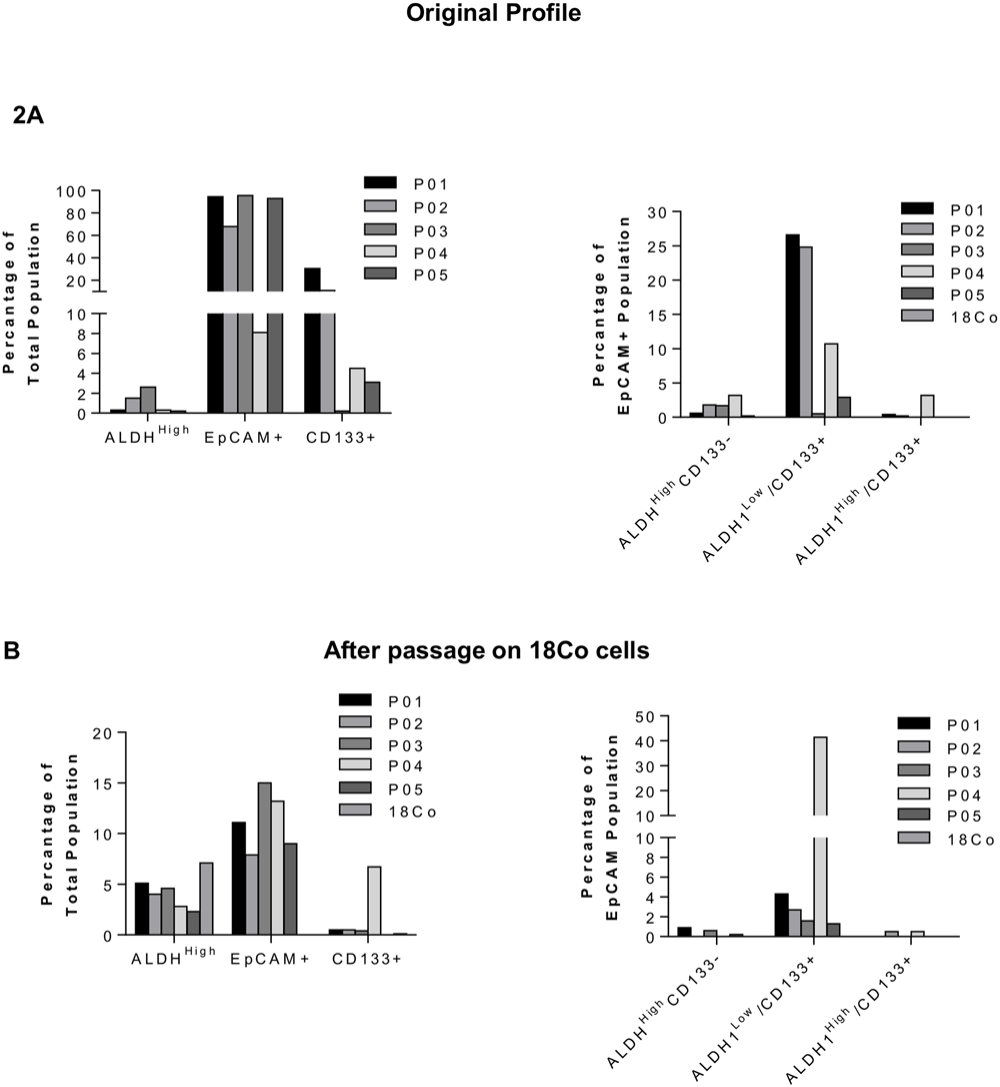
Flow cytometric profiling of spheroids under co-culture conditions. (A) Original sample profiles expressed as a percent of total cells, and as a percent of EpCAM^+^ cells. (B) Samples following one passage with 18Co cells expressed as a percent of total cells, and as a percent of EpCAM^+^ cells.

Fig. 2A depicts original flow cytometry profiles from five CRLM samples and 18Co cells, expressed both as a percent of the total cellular population and as a percent of the EpCAM^+^ population. The total EpCAM^+^ population was >75% in 4/5 samples, with ALDH^high^ consistently <4%. 18Co cells were EpCAM^-^. Co-culturing of tumour samples with 18Co monolayer cells resulted in 5 out of 5 different patient samples (100%) forming spheroids (S5 Fig) which could be successfully passaged. However, it was observed that the EpCAM^+^ epithelial cell population decreased from >75% of cells in the original tumour specimens to <15% in all five spheroid samples (Fig. 2B), yet total ALDH activity was increased. When the ALDH^high^ population was further analysed as a percentage of the EpCAM^+^ population, ALDH activity was comparable with that observed in the original tumor samples. 18Co cells alone expressed high ALDH activity and formed spheroids as consistently and reproducibly as CRLM samples cultured with 18Co cells. Flow cytometric analysis thus revealed that spheroids in co-culture consisted predominantly of 18Co fibroblasts, and that incorporation of 18Co cells into patient-derived spheroids was responsible for the high ALDH1 activity, erroneously intimating stem-like characteristics in this model.

### TIC marker expression and spheroid formation

**Fig 3:**
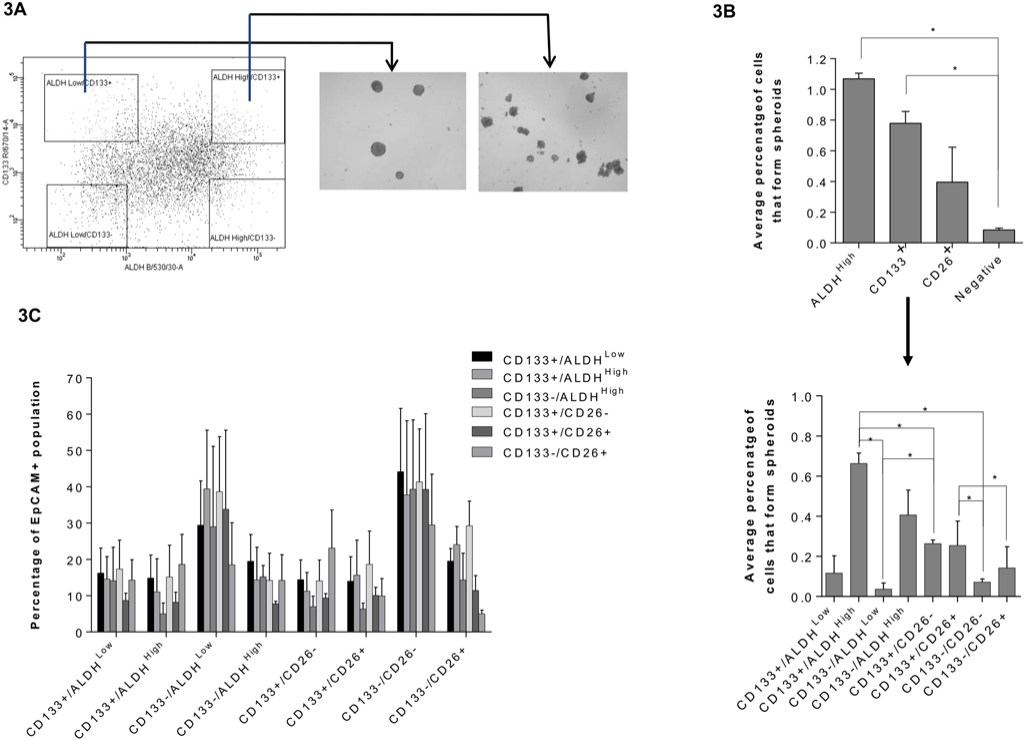
Analysis of the spheroid reforming ability of sorted spheroid TIC populations. (A) Gating strategy for cell sorting of samples based on TIC marker expression, and typical spheroid formation. (B) Mean percent of cells that form spheres from each population (N = 3 from a single patient sample, mean ± SEM, a paired sample t-test, * = P≤0.05). Spheroid forming ability was determined by the number of spheroids formed as a percentage of sorted cells. (C) Expression of TIC markers in spheroids grown from sorted spheroid TIC populations (N = 3. Mean ± SEM, paired sample t-test).

Spheroid forming ability for each population individually showed significantly higher spheroid formation in both the ALDH^high^ and CD133^+^ expressing populations compared to ALDH, CD133 and CD26 negative populations (Fig. 3A/B). Further stratification of these populations revealed CD133^+^/ALDH^high^ populations to have significantly greater spheroid forming ability than CD133^-^/ALDH^low^, CD133^+^/CD26^-^ and CD133^-^/CD26^-^ populations, with CD133^+^/CD26^+^ having a significantly higher spheroid forming ability than the CD133^-^/CD26^+^ population. This data was corroborated using a scoring system in which spheroid size, number and their ability to be passaged, was taken into account (S6 Fig). ALDH activity was the only marker individually which correlated positively with spheroid formation across samples (R^2^ = 0.42), with spheroid forming ability in the order of ALDH^high^>CD133^+^>CD26^+^.

Following spheroid formation and outgrowth, cells were sorted into all of the component populations for EpCAM, ALDH, CD133 and CD26, and spheroids from each population re-grown and re-analysed for expression of TIC markers (Fig. 3C). Here, no significant differences were observed, as each of the sorted populations were able reform every other population to a similar degree. This alludes to a high degree of TIC plasticity even in patient-derived samples.

### Effects of culture conditions on cellular plasticity

**Fig 4:**
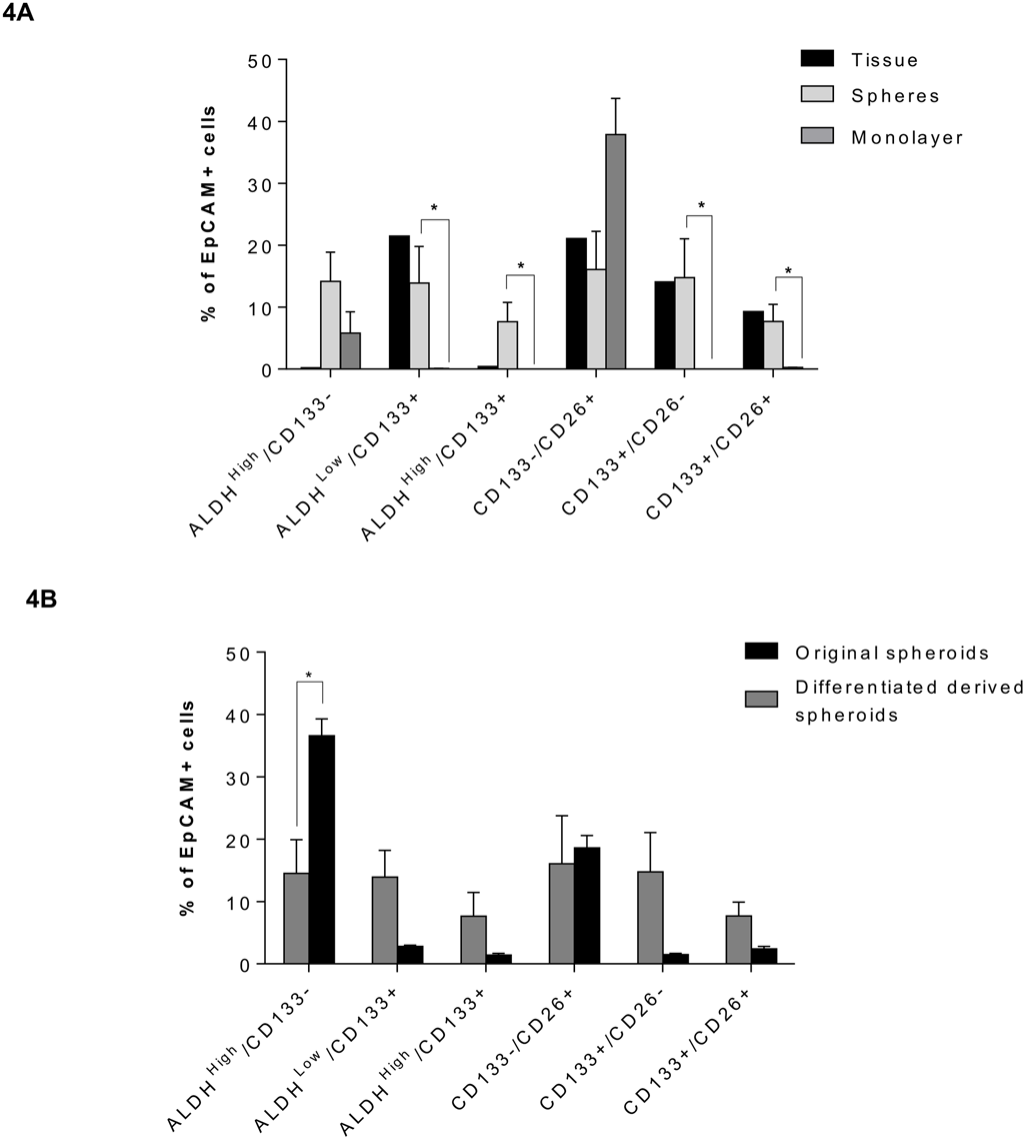
Comparison of TIC marker expression between an original tissue sample, matched spheroids and differentiated cells. (A) comparison of TIC marker expression between tissue, spheroids and differentiated cultures from the same patient (N = 1 for tissue (stats not shown), N = 4 for spheroids and N = 3 for differentiated cells, mean ± SEM, paired sample t-test, * = P≤0.05). (B) TIC marker expression comparing original spheroids with spheroids produced from differentiated cells when placed under low adherence conditions (N = 3, mean ± SEM, paired sample t-test, * = P≤0.05).

To examine whether culture conditions effect TIC marker expression in the CRLM model, expression of markers was compared between original tumor tissue, spheroids and differentiated spheroids grown on monolayer (all from the same patient). Culturing cells as spheroids caused an increase in the proportion of EpCAM^+^ ALDH^high^/CD133^-^ and ALDH^high^/CD133^+^ cells by 14% and 7% respectively, compared to tissue. CD133^+^ cells were not supported by differentiating culture conditions, with detection of CD133^+^-containing populations at negligible amounts only (Fig. 4A). Once spheroids had been allowed to differentiate, they were placed back under low adherence conditions to assess whether they still had the ability to reform spheroids. Differentiated cells were successfully able to reform spheroids, but had a significantly lower ALDH^high^/CD133^-^ population compared to the original spheroids (Fig. 4B). Changes to culture conditions (even for one passage) were clearly able to affect TIC profiles in CRLM-derived cells.

### Growth of CRLM in NOD-SCID mice

**Fig 5:**
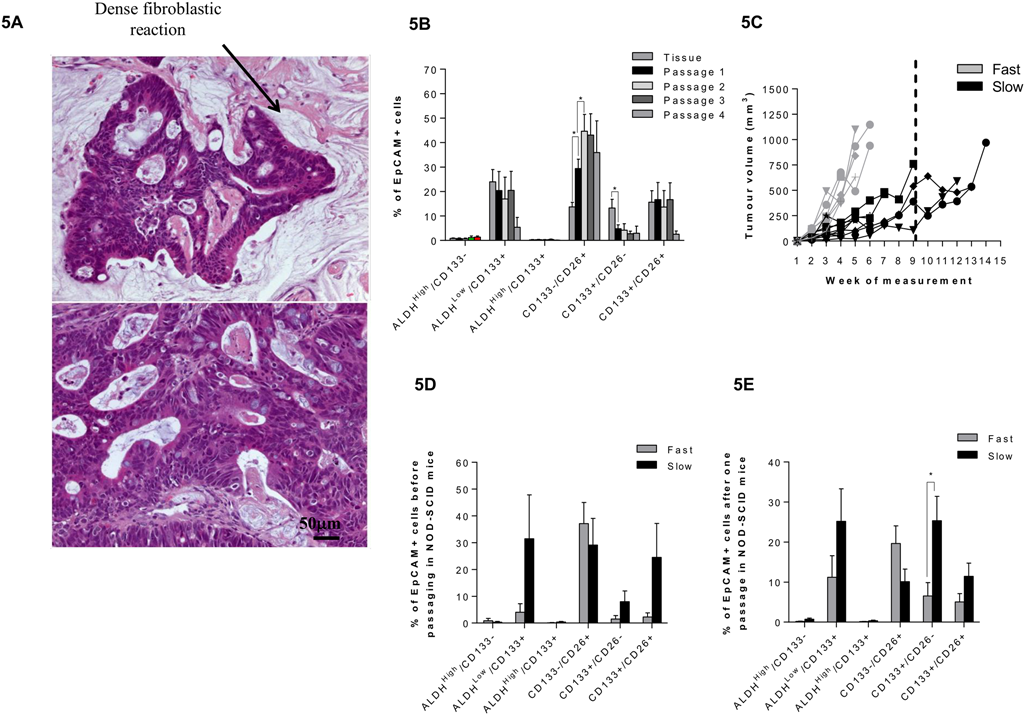
Serial passaging of CLRM tissue in NOD-SCID mice. (A) Tumor characterisation following engraftment of liver metastases into NOD-SCID mice. Haematoxylin/Eosin staining of the original hepatic metastatic lesion (upper panel) and tumor tissue following 2 passages in NOD-SCID mice (lower panel) (x20 objective). (B) Flow cytometric analysis of marker expression following each passage (Tissue N = 12, Passage 1 N = 11, Passage 2 N = 7, Passage 3 N = 6 and passage 4 N = 2, * = P≤0.05, t-test, mean ± SEM). (C) Tumor growth rates (passage 1) following the first NOD-SCID implantation (N = 11). (D) flow cytometric analysis of fast vs slow growing samples (directly from patient), (N = 5/group, mean ± SEM). (E) flow cytometric analysis of fast vs slow growing samples (following one passage), (N = 5/group, mean ± SEM, * = P≤0.05).

A further experimental procedure which allows maintenance of TIC populations is engraftment into NOD-SCID mice *via* subcutaneous injection of tumor tissue. Eleven of 15 (73%) CRLM samples were successfully engrafted into mice. Of the 15 patients from whom CRLM engraftment tissues were obtained, 11 were subject to previous chemotherapy and 4 were chemotherapy naïve. Growth of the xenografts was characterised by periods of 13000B003146 days from implantation until first detection of palpable tumors, and of a further 4300B003122 days until tumor harvest (tumors of ~17 mm/5% body weight). Following dissociation of xenografted tumor tissue, 9 out of 11 samples (82%) formed spheroids (>20 μm diameter) using standard low attachment conditions, compared to 0 out of 11 of these samples prior to NOD-SCID passage. Histological characteristics of the original tumor were maintained following serial xenografting with the only significant difference being greater fibroblastic reaction in the non-passaged tumours (Fig. 5A). Following one passage through NOD-SCID mice, CD133^-^/CD26^+^ expression was significantly increased, rising from 13.7% (±1.8%) in the original tumor tissue, to 29.4% (±3.8%) in xenograft tissue (Fig. 5B). Growth of the initial CRLM xenografts were then stratified in to slow-(> 9 weeks), or fast-growing tumors (< 9 weeks) (Fig. 5C) in order to observe whether TIC marker expression correlated with tumor growth rate. TIC marker analysis of the original tumor based upon xenograft growth rate, revealed a lower proportion of all CD133^+^-containing populations in the fast growing tumors compared to the slow growing ones (Fig. 5D). Following one passage through the NOD-SCID mice, a similar pattern was observed, with the CD133^+^/CD26^-^ population significantly lower in the fast growing tumors (Fig. 5E).

## Discussion

There is a need to better understand the TIC characteristics of cancers which have metastasised from their primary site of origin to distant organs capable of maintaining a metastatic niche [26]. It has been proposed that this niche is also capable of maintaining a TIC component that may play a role in resistance to neo-adjuvant or palliative chemotherapy. Therefore, metastatic TICs need to be better characterised in order to provide models which may have use in predicting efficacy of new drug regimens in late stage cancers. Metastatic colorectal cancer has been particularly poorly characterised in this respect.

The work described here assessed whether *ex vivo* CRLM models were able to maintain putative TIC populations which reflect those observed in the original tumour, as has been reported for primary CRC. Characterisation of TICs and the effects that isolation techniques and donor variation could have upon them, may play a crucial role in assessing future TIC-targeting strategies. In non-cancer settings, both age and gender dimorphisms have been shown to effect stem cells from a variety of origins, with steroid- and age–related effects governing both neuronal [27] and mesenchymal stem cell fate [28,29]. Age and gender appear to not only govern the relative expression of particular markers, but can also be responsible for a high degree of plasticity which will ultimately dictate terminal differentiation pathways. Whilst this is thought to be a critical determinant for potential utility of stem cells in stem cell therapy research, these effects have been disregarded in the cancer setting and the search for targeted TIC therapies. Within the study described here, age demographics for metastatic CRC (mCRC) were consistent with other studies [30], and so it is unsurprising that no correlations were observed between age and TIC marker expression. Female patients showed an increased expression in CD133 populations compared to their male counterparts. In endometrial cancers, androgen signalling/ receptor expression is shown to increase in chemotherapy resistant cells, and has been proposed to correlate with increased CD133 expression and TIC characteristics [31]. Circulating CD133^+^ endothelial progenitor cells were observed to be significantly increased in pre-menopausal women compared to males/post-menopausal women [32], although most of the female patients in the CRLM study described here would fall within the post-menopausal cohort. Involvement of androgen signalling in regulating stemness, however, remains ambiguous, as differentiation of embryonic stem cells is associated with induction of the progesterone receptor gene and concurrent increased signalling via this pathway [33]. The evidence for increased CD133 expression as a factor associated with poor prognosis in colorectal cancer (stages I-III) is accumulating [34,35], and it may indeed be associated with metastatic spread [5,36,37].

Contrary to much of the published literature (most notably in established CRC cell lines), chemotherapy had relatively few effects on CRLM TIC markers. Other reports in which stem-like components of CRLM have been investigated often fail to iterate whether patients have undergone chemotherapy at any point during the natural history of their disease. Several accounts allude to alterations in stem cell marker expression or the stem cell niche following chemotherapy [38,39]. It is becoming increasingly well documented that metastatic colonisation may require niche signalling from stromal components including mesenchymal, immune and vascular cells as well as a variety of extracellular matrix components [40,41] all of which could be subject to drug-induced changes. Once migratory TICs have reached a favourable metastatic niche, seeding occurs, whereupon they have the ability to recapitulate a heterogeneous tumor. In keeping with current practices within the UK National Health Service, many colorectal cancer patients will have undergone adjuvant chemotherapy following removal of the primary tumour, or neo-adjuvant chemotherapy to render liver metastases amenable to surgical resection. In the study described here, nearly three quarters of all patients (36/50) underwent chemotherapy at some point prior to resection of colorectal liver metastases. Chemotherapy caused a decrease in both the EpCAM^+^/ALDH^low^/CD133^+^ population, and the EpCAM^+^/CD133^+^/CD26^-^ population, observed almost exclusively in the male cohort, although this did not appear directly related to how recently the individuals received their chemotherapy, nor did it predict engraftment rate or spheroid formation. The reduced size of CD133^+^ populations following chemotherapy (rather than the increase observed in other cancers including rectal cancers and glioblastomas [42,43]) may lend further credence to support the notion that CD133^+^ is not the primary TIC marker in mCRC, as the CD133^+^ population in mCRC appears to be targeted by current standard care chemotherapy strategies. However, as CD133^+^ populations were significantly lower in the fast growing patient-derived xenografts, it suggests this component to be less proliferative and perhaps less well differentiated, contributing less to the overall tumor bulk than the CD133^-^ populations.

Currently, in most articles reporting propagation of colorectal metastases in the liver, intrasplenic/orthotopic injection of a variety of established human colorectal cancer cell lines (including HCT116, HT29, SW480 and SW48) has been used [44–46]. Other strategies rely upon extrapolation of invasive characteristics of established cell lines in *in vitro* systems in order to stratify colorectal cancer cells as ‘metastatic’, rather than deriving them experimentally from patients’ metastases [47,48]. However, it is well known that established cell lines often maintain little fidelity compared with that of the original tumour due to their inherent genetic instability during long-term passage [49]. Hence, establishment and characterisation of readily propagatable models derived directly from surgical specimens is essential to generate more clinically relevant platforms that can be used for the development of new therapeutic interventions or medicine stratification protocols.

The spheroid model has gained popularity as a 3D model which may better mimic tumor architecture and response to drug intervention than 2D culture. Growth of spheroids directly from patient-derived tissues is conceivably a way of propagating cellular populations which not only maintain the genetic heterogeneity of the patient cohort from which they are derived, but also provide a model that enriches for the TIC component, allowing more definitive assessment of TIC targeting through drug intervention. However, even here, ambiguity exists as to the absolute definition of the spheroid model, which may also affect how the models are defined by their TIC characteristics. Weiswald *et al*., [50] allude to this in their use of ‘colospheres’, derived directly from disggregated patient tissue, and ‘spheroids’, in which tumor cells were first grown as a monolayer with spheroids subsequently cultured under low attachment conditions.

Recently, Ray *et al*. [51] generated a xenograft model from one colorectal liver metastasis sample, which was first propagated as spheroid bodies prior to injection into nude SCID mice. However, data regarding spheroid formation efficiency or xenograft take rates are not described, and the results presented are for a single sample only. Pang *et al* [52] revealed CD26^+^ populations to represent a putative stem-like component within CRLM, which efficiently formed spheres when sorted and formed tumors when xenografted into NOD-SCID mice. In this study, spheroid formation rates from patient-derived metastases were not reported, but spheroids were cultured under standard low attachment conditions. Following this report, we went on to investigate CD26 expression both pre- and post- tissue engraftment in the NOD-SCID mice. Whilst none of the original patient-derived samples formed true spheroids under low attachment conditions, following passage in the NOD-SCID mouse, spheroid forming ability was significantly enhanced. ALDH^high^ activity appeared to be the best predictor of spheroid forming ability, but the average ALDH^high^ population observed in tissue was 0.6%, with many patients (27%) having no observable activity. ALDH^high^ activity was increased following spheroid formation under conditions that are proposed to maintain a stem-like phenotype. However, this intimates that conditions were sufficient to engender such plasticity within the cells that they began to express stem cell markers that previously were at, or below the limits of detection. Similarly for CD26, levels appeared to increase following passage in the NOD-SCID mice, despite the overarching pathology of the PDX tumors remaining the same as the original patient tumors. This gives rise to further thought that TIC characteristics following propagation will differ from original patient samples in the CRLM TIC model, conceivably due to the inevitable micro-environmental changes. This degree of cellular plasticity would therefore suggest that it is even more critical to utilise tissue-derived samples, rather than established cell lines, when investigating TIC expression across a variety of pathologies. Furthermore, *ex vivo* tissue cultures should be used at a low a passage as possible, with culture conditions carefully controlled. Further issues in *ex vivo* model choice must take into account that spheroid forming ability may not always be driven by the epithelial TICs under investigation. Stromal cells with fibroblastic phenotypes express many of the markers associated with TICs, and are able to efficiently form spheroids in their own right. Effective strategies to remove contaminating components must be therefore be employed for confident analysis of TIC data. This is not to say however, that *ex vivo* models do not provide a useful adjunct for assessing potential utility of therapeutic interventions, rather that caution should be applied if ultimately models are subsequently utilised to dictate personalised therapy options.

Other markers that have been suggested to be crucial in sphere forming ability and ‘stemness’ in colorectal liver metastases include expression of Oct 3/4 and Nanog [53], which again remained unaltered following chemotherapy in the study described here. It must therefore be considered that there are, as yet unrecognised or hitherto unpublished TIC markers that may define metastatic TICs much better than those extrapolated from characterisation of the primary tumor. This presents a plausible scenario, as it is recognised that TIC phenotypes may differ even between tumors of the same subtype, and that TICs derived from the metastatic population may indeed develop their own distinct phenotype from that of the primary tumour [54]. However, this observation is also likely to have dependency upon the tissue of origin [55], whether there has been any pre-operative chemotherapy and propagation methods utilised for TIC sub-culture.

Increasingly, patient tissue is being used to generate suitable models for testing novel anti-cancer interventions. However, there is often little information as to the success rates of these models, generating an unrealistic ‘one model fits all’ paradigm. Within the study described here, we give insight into issues which could arise from model selection, patient gender, chemotherapy reporting, NOD-SCID engraftment rates and spheroid forming capacity across our mCRC CRLM patient cohort. It appears that selection and reporting of appropriate models could impact not only upon the TIC markers characterised on a patient by patient basis, but could also have an impact on the future direction of TIC-targeting strategies. Such strategies must be based upon a majority population, rather than the oft small cohorts which readily allow for easy propagation. This is essential to enable mechanistic and interventional studies to be performed across the majority of samples obtained, in order to maintain population heterogeneity and circumvent potential bias caused by experimental limitations.

## Supporting Information

S1 Fig Association of TIC marker expression with age. To assess whether age is associated with TIC marker expression, a scatter plot of age vs expression was performed using a line of best fit, with the R^2^ value indicating correlation: EpCAM N = 62, CD133 N = 62, ALDH N = 62, CD26 N = 44.
(TIFF)

S2 Fig Association of TIC marker expression with gender and chemotherapy. Expression of TIC markers were stratified by gender and whether the patient had received chemotherapy. N = 6, 12, 8 and 23 for female chemo-naïve and treated, and male chemo-naïve and treated respectively, when analysing ALDH combinations. For CD26 combinations N = 5, 8, 7 and 12 for female chemo-naïve and treated, and male chemo-naïve and treated respectively. Error bars represent SEM, and * = P<0.05.
(TIFF)

S3 Fig Expression of a panel of pluripotent stem cell markers in CRLM EpCAM+ cells stratified by TIC marker expression. The proteome profiler array was carried out on a total of 23 patients, with the top three and lowest three expressing samples for each marker being averaged and compared (except for ALDH^low^ activity as six patients had no activity and so were averaged together), (A): EpCAM, (B): CD133, (C): ALDH and (D): CD26. Additionally, the proteome profiler data for chemo-naïve (N = 2) or chemo-treated (N = 13) patients was averaged and compared, (E). Error bars represent SEM.
(TIFF)

S4 Fig The effects of growing single cells in media supplemented with growth factors, or in matrigel. (A) Demonstrates the lack of growth promotion provided by culturing CRLM cells in media supplemented with R-spondin, Wnt3A and HGF. The same results were seen when cells were grown embedded in matrigel (B), using the standard spheroid culturing media.
(TIFF)

S5 Fig Light microscopy images of spheroids produced by co-culture of tumor cells on 18Co cells. Arrow depicts the fibroblast extensions produced by the 18Co cells. Imaged using a X20 objective.
(TIFF)

S6 Fig Correlation of TIC marker expression with spheroid growth capacity in patient samples. To see whether expression of TIC markers was associated with spheroid growth, spheroids were correlated with expression levels against a designated score that depended on the number and size of spheroids. The scoring system was; 0 = no spheroids, 1 = less than 5 spheroids, 2 = small and 5–10 spheroids, 3 = large and 5–10 spheroids, 4 = large and over 10 spheroids which were able to be passaged. The expression levels for TIC markers in the parent tissue was then correlated with spheroid score. For the scores 0, 1, 2, 3 and 4 the respective N numbers are; 12, 6, 2, 3 and 3, except EpCAM which has a respective N numbers of 12, 3, 2, 2 and 1.
(TIFF)
