# Supplementary figures and images for: Characterization and Propagation of Tumor Initiating Cells Derived from Colorectal Liver Metastases: Trials, Tribulations and a Cautionary Note
Source: PLoS One. 2015 Feb 6;10(2):e0117776. doi: 10.1371/journal.pone.0117776 (PMC4319830; doi:10.1371/journal.pone.0117776)

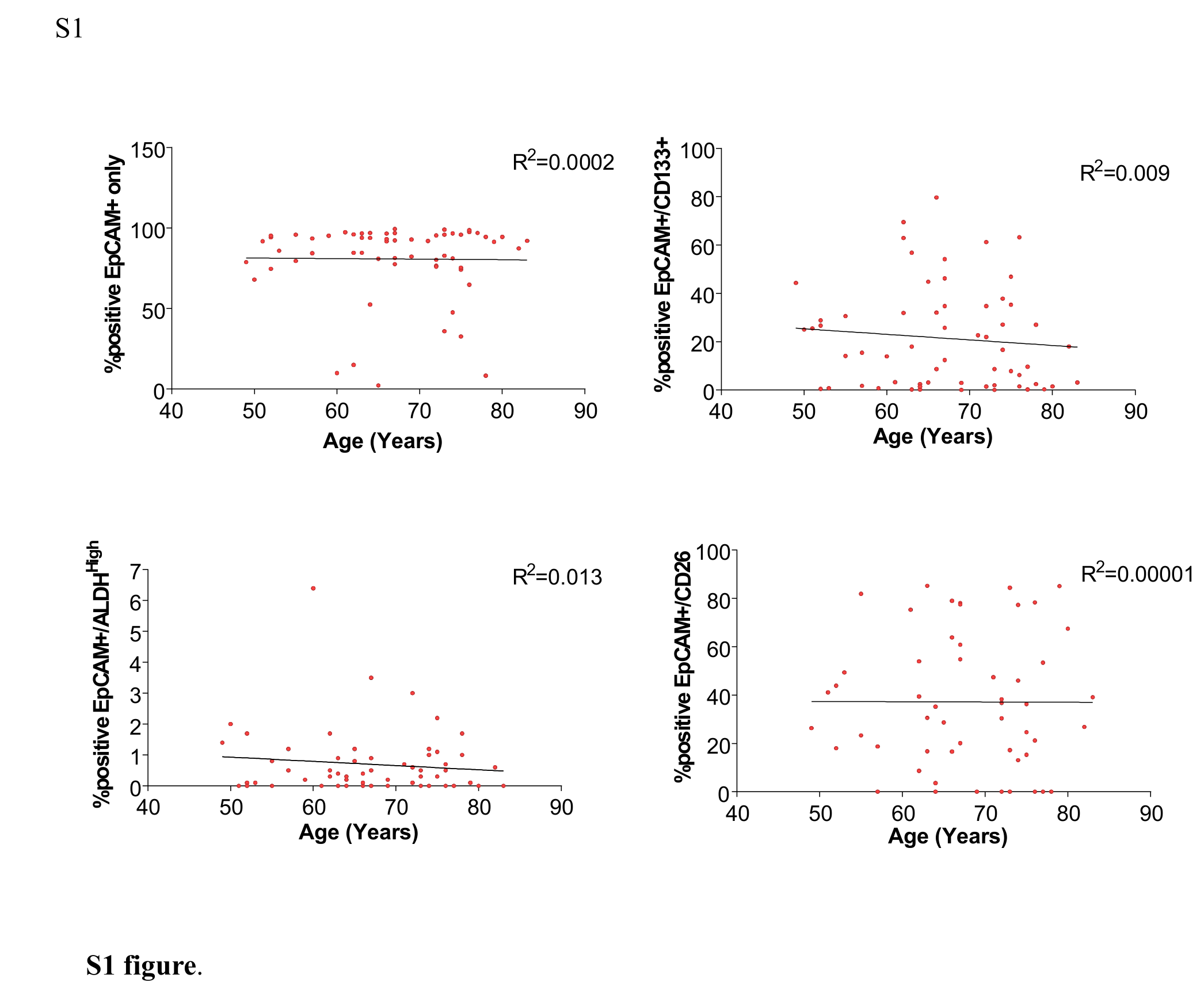

Supplement: S1 Fig — To assess whether age is associated with TIC marker expression, a scatter plot of age vs expression was performed using a line of best fit, with the R2 value indicating correlation: EpCAM N = 62, CD133 N = 62, ALDH N = 62, CD26 N = 44. (TIFF) [file pone.0117776.s001.tiff]

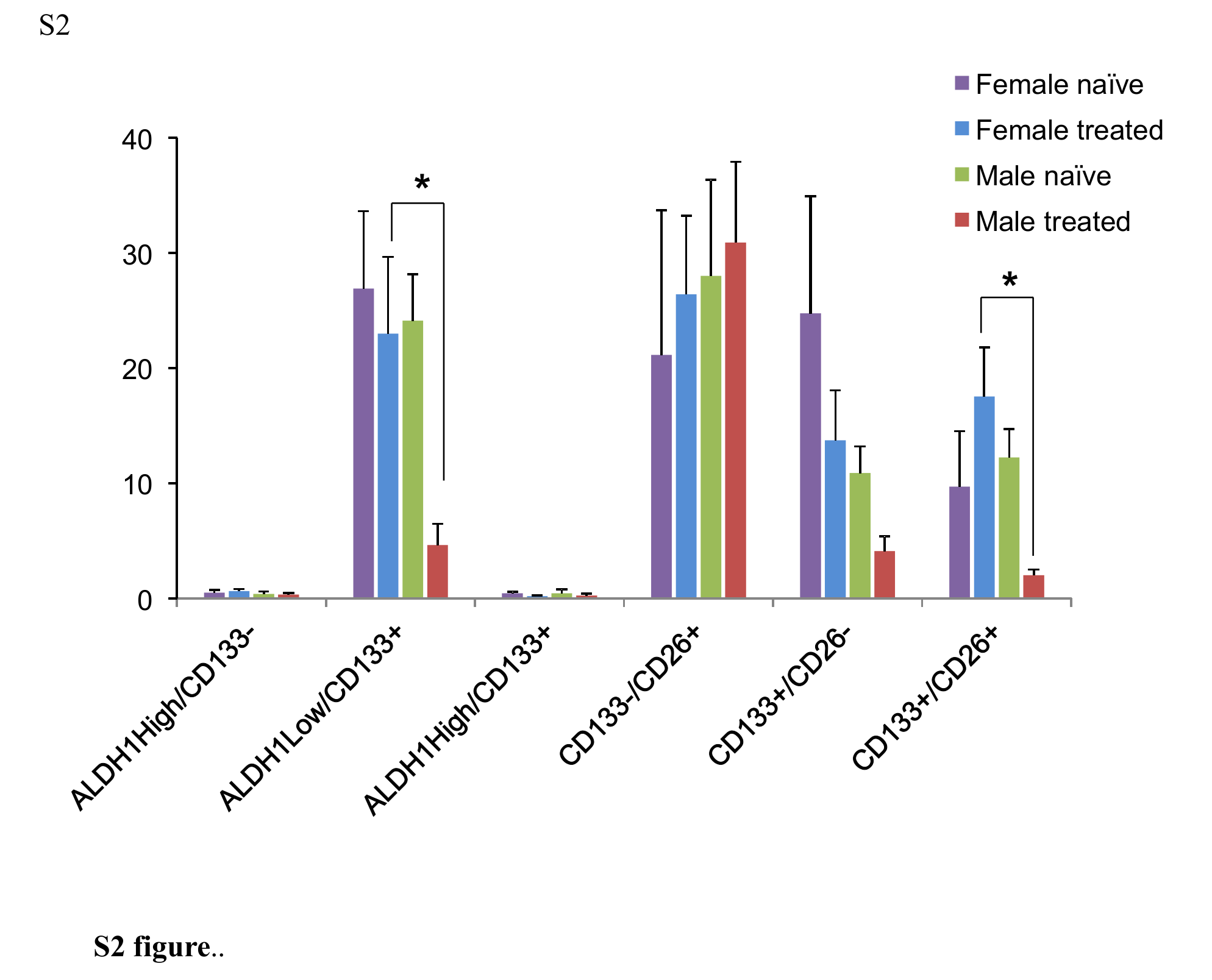

Supplement: S2 Fig — Expression of TIC markers were stratified by gender and whether the patient had received chemotherapy. N = 6, 12, 8 and 23 for female chemo-naïve and treated, and male chemo-naïve and treated respectively, when analysing ALDH combinations. For CD26 combinations N = 5, 8, 7 and 12 for female chemo-naïve and treated, and male chemo-naïve and treated respectively. Error bars represent SEM, and * = P<0.05. (TIFF) [file pone.0117776.s002.tiff]

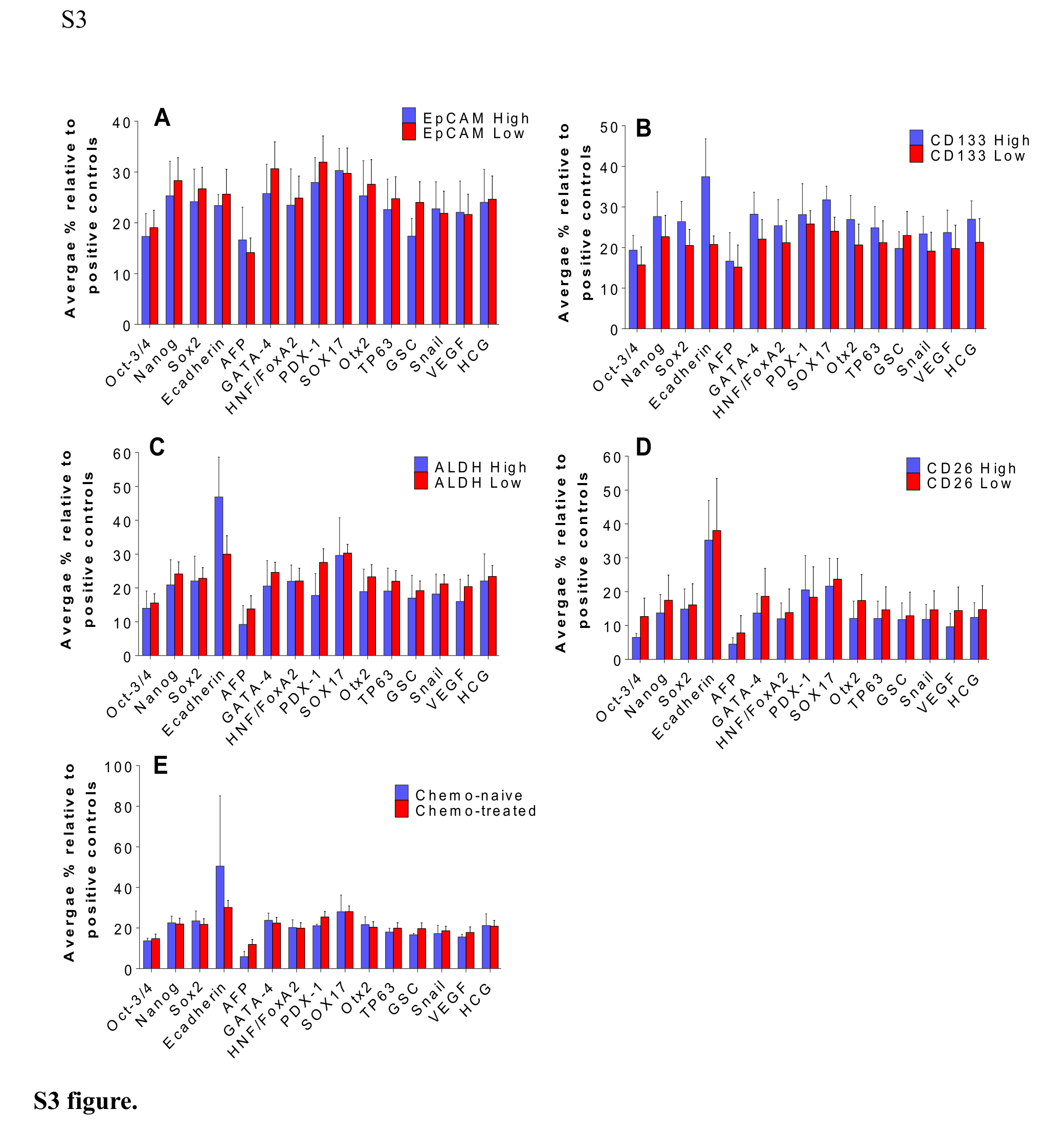

Supplement: S3 Fig — The proteome profiler array was carried out on a total of 23 patients, with the top three and lowest three expressing samples for each marker being averaged and compared (except for ALDHlow activity as six patients had no activity and so were averaged together), (A): EpCAM, (B): CD133, (C): ALDH and (D): CD26. Additionally, the proteome profiler data for chemo-naïve (N = 2) or chemo-treated (N = 13) patients was averaged and compared, (E). Error bars represent SEM. (TIFF) [file pone.0117776.s003.tiff]

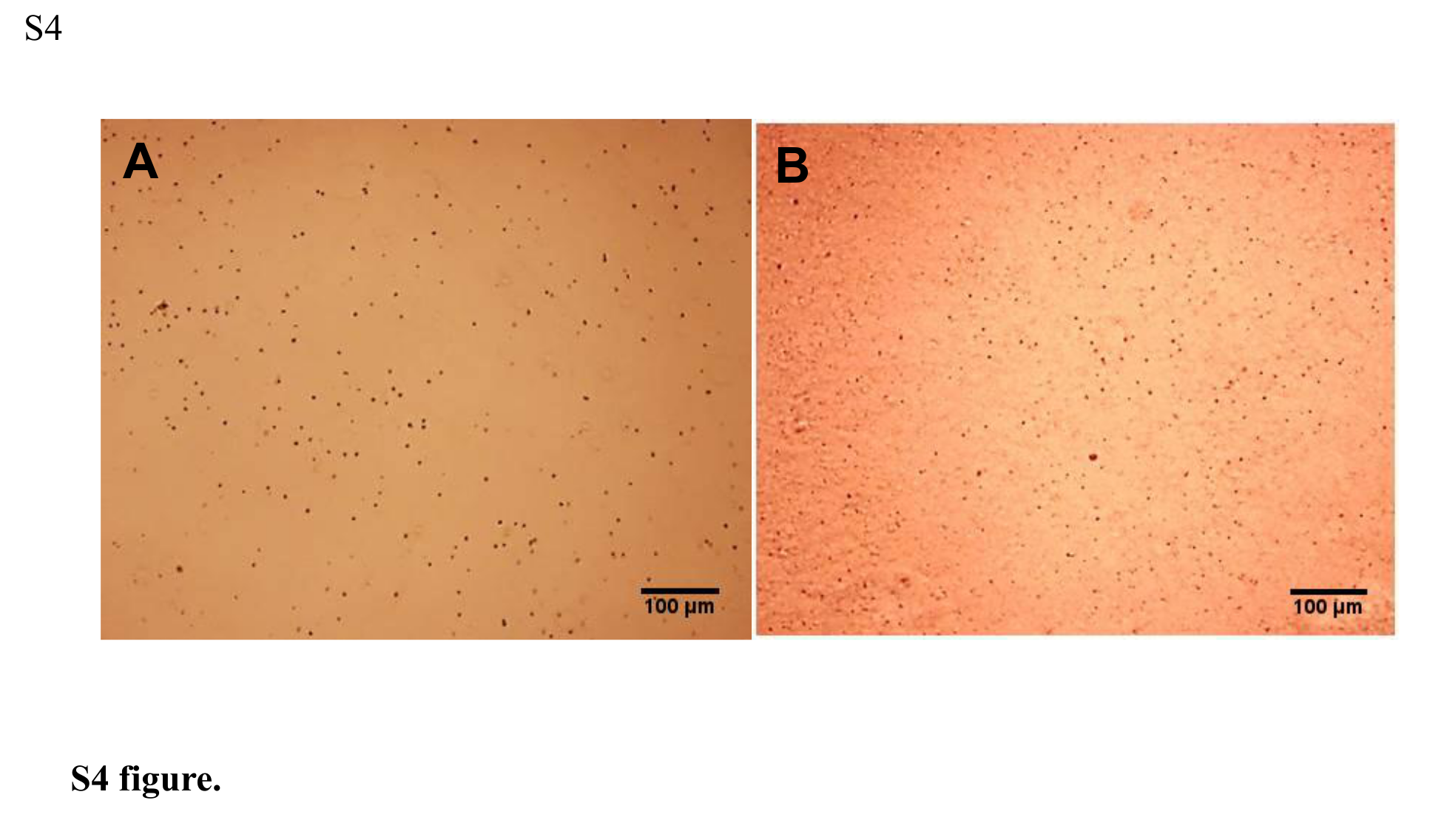

Supplement: S4 Fig — (A) Demonstrates the lack of growth promotion provided by culturing CRLM cells in media supplemented with R-spondin, Wnt3A and HGF. The same results were seen when cells were grown embedded in matrigel (B), using the standard spheroid culturing media. (TIFF) [file pone.0117776.s004.tiff]

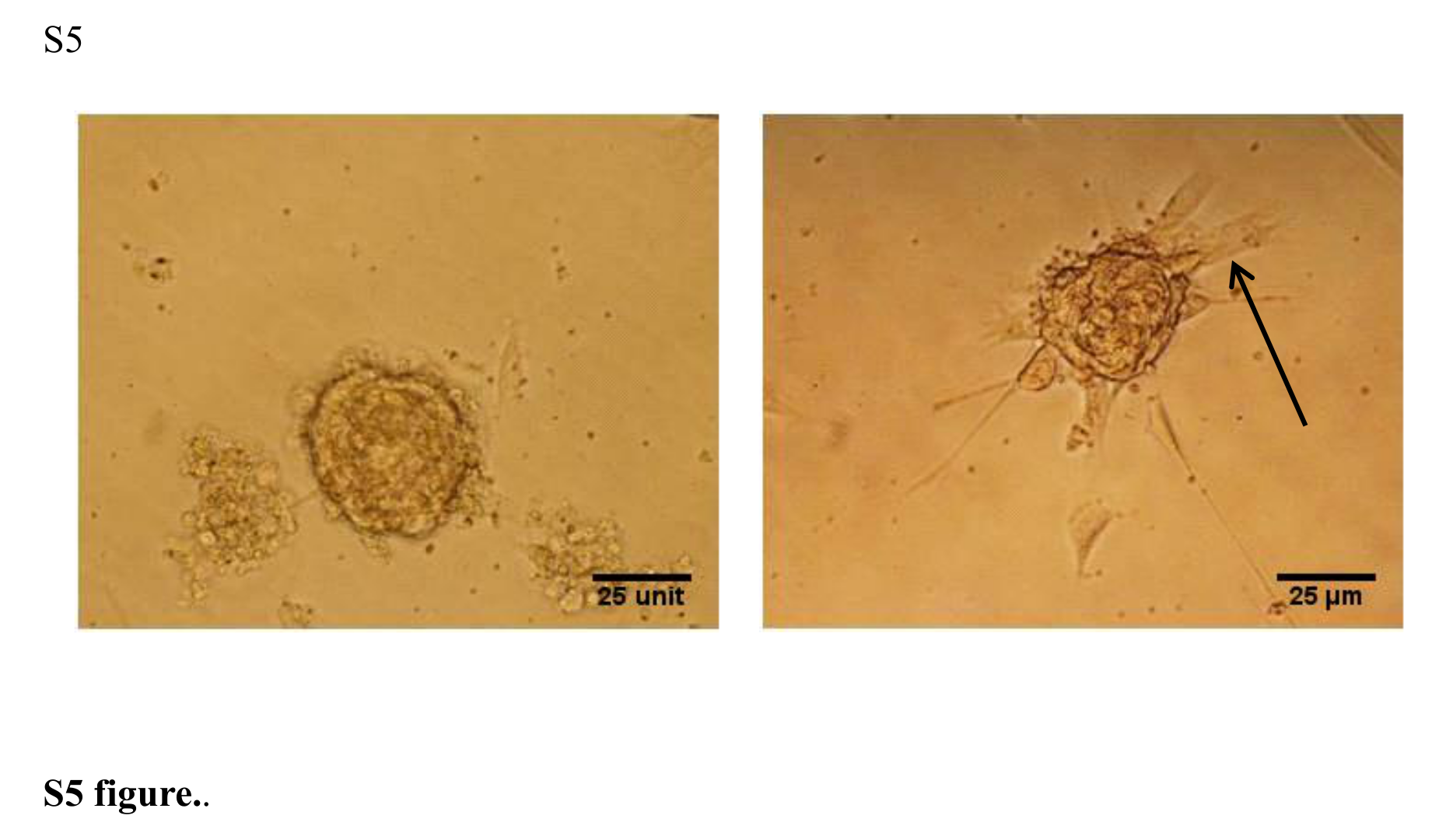

Supplement: S5 Fig — Arrow depicts the fibroblast extensions produced by the 18Co cells. Imaged using a X20 objective. (TIFF) [file pone.0117776.s005.tiff]

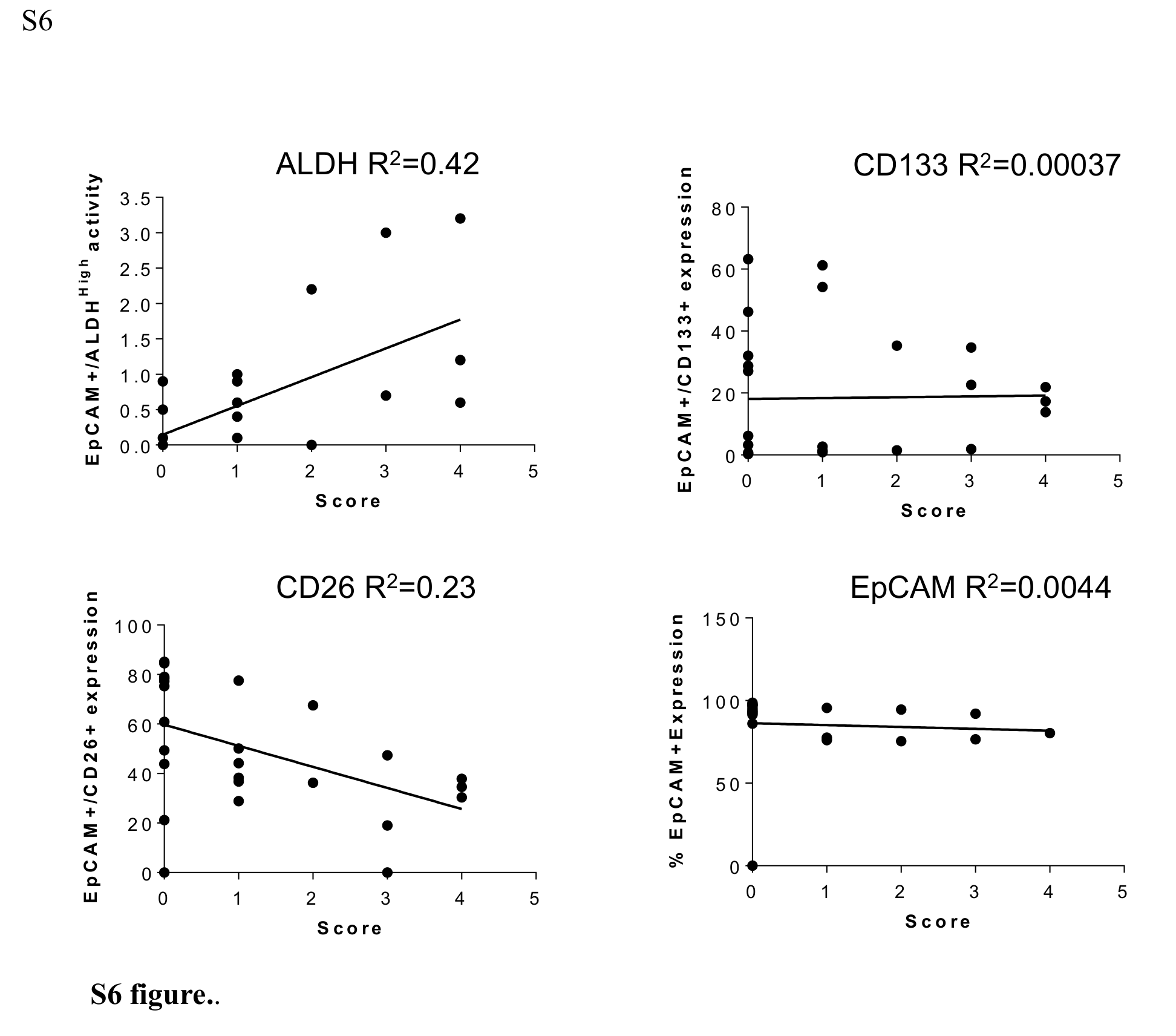

Supplement: S6 Fig — To see whether expression of TIC markers was associated with spheroid growth, spheroids were correlated with expression levels against a designated score that depended on the number and size of spheroids. The scoring system was; 0 = no spheroids, 1 = less than 5 spheroids, 2 = small and 5–10 spheroids, 3 = large and 5–10 spheroids, 4 = large and over 10 spheroids which were able to be passaged. The expression levels for TIC markers in the parent tissue was then correlated with spheroid score. For the scores 0, 1, 2, 3 and 4 the respective N numbers are; 12, 6, 2, 3 and 3, except EpCAM which has a respective N numbers of 12, 3, 2, 2 and 1. (TIFF) [file pone.0117776.s006.tiff]
